# Association of 24 h–systolic blood pressure variability and cardiovascular disease in patients with obstructive sleep apnea

**DOI:** 10.1186/s12872-017-0723-y

**Published:** 2017-12-06

**Authors:** Xiao Ke, Yan Sun, Rongfeng Yang, Jiawen Liang, Shaoyun Wu, Chengheng Hu, Xing Wang

**Affiliations:** 1 Department of Cardiology, Shenzhen Sun Yat-sen Cardiovascular Hospital, Dongmen North Road 1021, Shenzhen, 518112 China; 2 Department of Endocrinology, Xili People’s Hospital of Nanshan District, Shenzhen, 518000 China; 3 Department of Cardiology, The First Affiliated Hospital of Sun Yat-sen University, 58 Zhongshan Road 2, Guangzhou, 510080 China

**Keywords:** Obstructive sleep apnea, Blood pressure variability, Cardiovascular diseases

## Abstract

**Background:**

To evaluate association of 24 h–systolic blood pressure (SBP) variability and obstructive sleep apnea (OSA) as defined by the apnea-hypopnea index ≥5/h; and association of 24 h–SBP variability and prevalent cardiovascular disease (CVD) in OSA patients.

**Methods:**

Participants underwent polysomongraphy to evaluate the presence of OSA, and 24 h–ambulatory blood pressure monitoring was applied to evaluate 24 h–SBP variability as indexed by weighted 24 h–standard deviation (SD) of SBP. Between-group differences were evaluated in participants with and without OSA. Participants with OSA were divided into high and low 24 h–SBP variability groups and between-group differences were evaluated.

**Results:**

Mean age of 384 participants was 50 years old and 42.2% had OSA. Mean 24 h–systolic/diastolic BP were 130/78 mmHg, with mean weighted 24 h–SD of systolic/diastolic BP were 12.9/7.3 mmHg. Compared to those without OSA, OSA participants had higher clinic-, 24 h-, daytime- and nighttime-SBP, and weighted 24 h, daytime- and nighttime-SD of SBP. Age, prevalent CVD and OSA, usage of angiotensin converting enzyme inhibitor/angiotensin receptor blocker, calcium channel blocker and diuretic were significantly associated with 24 h–SBP variability. In OSA patients, compared to those with low variability, participants with high variability had higher weighted 24 h, daytime- and nighttime-SD of SBP. After adjusted for covariates including clinic-SBP and 24 h–SBP, per 1-SD increment weighted 24 h–SD of SBP was associated with 21% increased prevalent CVD.

**Conclusions:**

Patients with newly-diagnosed OSA have higher 24 h–SBP variability compared to those without OSA; in OSA patients, increased 24 h–SBP variability is associated with increased prevalence of CVD.

## Background

Hypertension is a well-known risk factor for cardiovascular diseases (CVD) and all-cause mortality [1, 2]. Most previous clinical trials used systolic blood pressure (SBP) as the therapeutic target and showed that decreasing SBP was beneficial for improving cardiovascular outcomes [3, 4]. After the publication of the SPRINT trial [5], the optimal SBP target for patients with hypertension has once again underwent intensive debate. Notably, BP is featured by high variability whereby the therapeutic BP target is debatable [6]. Importantly, prior observational studies and meta-analysis indicate that other than SBP level, SBP variability was also independently associated with cardiovascular outcomes [7–10].

Obstructive sleep apnea (OSA) is another major risk factor for CVD and cardiovascular mortality [11, 12]. In addition, prior prospective cohort studies demonstrate that patients with OSA have an increased incidence of hypertension compared to those without OSA [13, 14]. The underlying mechanisms are multifactorial including endothelial dysfunction, increased sympathetic output and arterial stiffness [15]. Notably, these pathological alterations are also associated with BP variability. Therefore, one may anticipate that OSA patients may have higher BP variability compared those without OSA; in addition, regarding the association of SBP variability and CVD, it may be possible that OSA patients with high SBP variability may have higher prevalence of CVD compared to their OSA counterparts with low SBP variability.

Nevertheless, up till now, few studies have investigated these aspects in patients with OSA. Therefore, using a cross-sectional design, we plans to evaluate whether OSA is independently associated with 24 h–SBP variability; in addition, in patients with OSA, whether 24 h–SBP variability is associated with prevalence of CVD.

## Methods

### Participants’ enrolment

Written informed consents were obtained from studied participants before recruitment and current study was approved by the Research Ethic Committee of Shenzhen Sun Yat-sen Cardiovascular Hospital. All participants were treated in accordance to the Declaration of Helsinki. Included criteria were as follows: participants who have documented essential hypertension and agreed to have polysomnography (PSG) evaluation. Excluded criteria were as follows: those who have a diagnosis OSA before or already have been treated with continuous positive airway pressure (CPAP), have documented diabetes mellitus and chronic kidney disease, and have ischemic stroke, myocardial infarction, or congestive heart failure in the past 12 months.

### Data collection

Demographic and clinical data including age, gender, smoking status, previous medical history and current medications usage were collected by using self-administered questionnaire with the help of investigators; anthropometric data including body mass index (BMI, calculated by weight in kilograms divided by height in squared meters), systolic/diastolic BP (SBP/DBP) and heart rate at rest were measured (HEM7200, Omron Healthcare, Tokyo, Japan) by investigators in accordance to the guideline recommendation [16]. In brief, BP was measured 3 times and the last two BP readings were averaged for the clinic-SBP/DBP. Overnight fasting venous blood was drawn for serum fasting plasma glucose (FPG), lipid profiles, high-sensitivity C-reactive protein (Hs-CRP) and creatinine (Cr) measurements. Cardiovascular disease included coronary heart disease (CHD) which was diagnosed based on clinical symptoms and coronary angiography or computer tomography with contrast, and ischemic stroke which was diagnosed based on clinical symptoms and signs and computer tomography or magnetic resonance imaging, and transient ischemic attack were excluded.

### Variability of BP evaluation

Variability of 24 h–SBP were assessed using 24 h ambulatory blood pressure monitoring (24 h–ABPM, The Spacelabs 90,217, Spacelabs Inc., Redmond, Wash), which was conducted in accordance to the European Society Hypertension practice guideline for ABPM [17]. Daytime and nighttime intervals were determined using sleep time reported by patients, and at least 20 valid awake and 7 valid asleep measurements should be recorded. The weighted standard deviation (SD) of 24 h–SBP was used as the variability of 24-SBP.

### OSA evaluation

OSA evaluation was performed using PSG (Philips) and those with central sleep apnea were excluded. According to the AASM guideline [18], airflow total blockage for >10 s, or >50% reduction in respiratory airflow with >3% reduction in saturation of arterial oxygen (SaO_2_) for >10 s is defined as apnea or hypopnea events, and the apnea-hypopnea index (AHI) is calculated by the total number of apnea and hypopnea events per sleep hour, with AHI ≥ 5 events/h was diagnosed as OSA [18].

### Statistical analysis

Continuous variables were expressed as mean ± SD and between-group differences were evaluated by the independent Student *t* test. Categorical variables were expressed as number and frequency of cases, and between-group differences were assessed by the Chi-square analysis or Fisher exact test. Association of weighted 24 h–SD of SBP and parameters of interest were evaluated using the linear regression analysis. Logistic regression analysis was applied to evaluate the association of weighted 24 h–SD of SBP and prevalent CVD. Statistical analysis was conducted by the SPSS 17.0 software (SPSS Inc., Chicago, USA).

## Results

### General characteristics

From June of 2016 to June of 2017, a total of 393 participants were enrolled from outpatient clinic. Among them, 5 patients could not finish 24 h ABPM and 4 patients subsequently diagnosed as central sleep apnea were excluded, therefore, 384 participants were included into final analysis. No significant differences in clinical characteristics between the excluded patients and included participants were observed. Overall, the mean age was 50 years old and male participants accounted for 64.1%. The mean AHI was 8.3 events/h and 42.2% of participants were diagnosed as OSA. The mean clinic-SBP/DBP were 141/86 mmHg, and the mean 24 h–SBP/DBP were 130/78 mmHg, with mean weighted 24 h–SD of SBP/DBP were 12.9/7.3 mmHg.

### Clinical characteristics comparisons between participants with and without OSA

As shown in the Table 1, the mean age of OSA patients was 53 years old, male participants accounted for 65.4%, 48.1% were current cigarette smokers, 40.7% had prevalent CVD and the mean number of anti-hypertensive medications was 2.5. Compared to those without OSA, participants with OSA were more elderly, had higher BMI, proportion of smokers and serum Hs-CRP level, and also had higher prevalence of CVD. The mean number of anti-hypertensive medications and clinic-SBP (143 ± 18 mmHg vs 139 ± 15 mmHg) were also significantly higher.

**Table 1:** Characteristics comparison between participants with and without OSA

Variables	Without OSA (*n* = 222)	OSA (*n* = 162)
Age (years)	47 ± 12	53 ± 15*
Male, *n* (%)	140 (63.1)	106 (65.4)
BMI (kg/m^2^)	23.3 ± 5.5	27.8 ± 6.8*
Current smoking, n (%)	78 (35.1)	78 (48.1)^#^
Clinic-SBP (mm Hg)	139 ± 15	143 ± 18*
Clinic-DBP (mm Hg)	85 ± 9	86 ± 10
Clinic-HR (bpm)	75 ± 10	79 ± 12
FPG (mmol/L)	5.6 ± 0.4	5.5 ± 0.6
TC (mmol/L)	5.0 ± 0.7	5.2 ± 0.5
TG (mmol/L)	1.8 ± 0.6	1.9 ± 0.8
HDL-C (mmol/L)	1.0 ± 0.4	1.1 ± 0.4
LDL-C (mmol/L)	3.2 ± 0.4	3.4 ± 0.6
Cr (umol/L)	74.9 ± 12.8	71.8 ± 13.6
Hs-CRP (mg/dL)	7.2 ± 3.6	9.7 ± 4.8*
AHI (events/h)	3.4 ± 1.4	11.6 ± 6.2^#^
CVD, *n* (%)	79 (35.6)	66 (40.7)*
Anti-platelet agent, n (%)	98 (44.1)	68 (42.0)
Statins, *n* (%)	79 (35.6)	59 (36.4)
ACEI/ARB, *n* (%)	160 (72.1)	119 (73.5)
CCB, *n* (%)	42 (18.9)	33 (20.4)
Diuretic, *n* (%)	70 (31.5)	52 (32.1)
Beta-blocker, *n* (%)	43 (19.4)	37 (22.8)
Alpha-blocker, *n* (%)	12 (5.4)	10 (6.2)
Anti-HTN medications number	1.7 ± 0.5	2.5 ± 0.8*

BMI = body mass index; SBP/DBP = systolic/diastolic blood pressure; HR = heart rate; bpm = beat per minute; FPG = fasting plasma glucose; TC = total cholesterol; TG = triglyceride; HDL-C = high density lipoprotein-cholesterol; LDL-C = low density lipoprotein-cholesterol; Cr = creatinine; Hs-CRP = high sensitivity C-reactive protein; SD = standard deviation; AHI = apnea hypopnea index; OSA = obstructive sleep apnea; CVD = cardiovascular disease; ACEI/ARB = angiotensin converting enzyme inhibitor/angiotensin receptor blocker; CCB = calcium channel blocker; *P < 0.05 and ^#^ *P* < 0.01 vs without OSA group

### 24 h BP parameters comparisons between participants with and without OSA

As presented in the Table 2, compared to those without OSA, participants with OSA had significantly higher 24 h–SBP (134 ± 14 mmHg vs 128 ± 12 mmHg) and weighted 24 h–SD of SBP (14.2 ± 4.6 mmHg vs 10.3 ± 3.1 mmHg). In addition, the daytime and nighttime SBP, weighted daytime- and nighttime-SD of SBP were also significantly higher in OSA group versus without OSA group. No statistical significances in parameters of DBP were observed.

**Table 2:** 24 h BP parameters comparisons between participants with and without OSA

Variables	Without OSA (*n* = 222)	OSA (*n* = 162)
24 h–SBP (mm Hg)	128 ± 12	134 ± 14*
24 h–DBP (mm Hg)	76 ± 9	79 ± 11
24 h–HR (beat per minute)	71 ± 10	73 ± 9
Weighted 24 h–SD of SBP (mm Hg)	10.3 ± 3.1	14.2 ± 4.6*
Weighted 24 h–SD of DBP (mm Hg)	6.8 ± 1.7	7.7 ± 2.0
Daytime-SBP (mm Hg)	133 ± 13	139 ± 16*
Daytime-DBP (mm Hg)	80 ± 10	83 ± 12
Daytime-HR (beat per minute)	77 ± 13	79 ± 14
Weighted daytime-SD of SBP (mm Hg)	12.5 ± 3.4	16.4 ± 4.9*
Weighted daytime-SD of DBP (mm Hg)	7.7 ± 1.8	8.3 ± 2.5
Nighttime-SBP (mm Hg)	122 ± 10	129 ± 12*
Nighttime-DBP (mm Hg)	71 ± 7	74 ± 9
Nighttime-HR (beat per minute)	65 ± 8	66 ± 8
Weighted nighttime-SD of SBP (mm Hg)	9.1 ± 1.4	12.6 ± 2.5*
Weighted nighttime-SD of DBP (mm Hg)	6.1 ± 1.4	7.0 ± 1.8

*P < 0.05 vs without OSA group; SD = standard deviation; SBP/DBP = systolic/diastolic blood pressure; HR = heart rate; bpm = beat per minute

### Association of weighted 24 h–SD of SBP and parameters of interest

Linear regression analysis was applied to evaluate whether the presence of OSA and other parameters of interest were independently associated with 24 h SBP variability. As presented in the Table 3, age, presence of CHD and OSA, and usage of angiotensin converting enzyme inhibitor/angiotensin receptor blocker (ACEI/ARB) were positively associated with weighted 24 h–SD of SBP; and usage of calcium channel blocker (CCB) and diuretic were negatively associated with weighted 24 h–SD of SBP.

**Table 3:** Association of weighted 24 h–SD of SBP and parameters of interest

Independent variables	Coefficient	SE	*P* value
Age (years)	1.683	0.254	<0.05
Male (vs female)	1.042	0.068	NS
BMI (kg/m^2^)	1.064	0.029	NS
Smoking (yes vs no)	1.109	0.027	NS
Hs-CRP (mg/dL)	1.043	0.035	NS
CVD (yes vs no)	1.842	0.418	<0.05
ACEI/ARB (yes vs no)	1.523	0.126	<0.05
CCB (yes vs no)	−1.753	0.195	<0.05
Diuretic (yes vs no)	−1.554	0.230	<0.05
Beta-blocker (yes vs no)	1.049	0.038	NS
Alpha-blocker (yes vs no)	−1.103	0.065	NS
OSA (yes vs no)	1.845	0.506	<0.05

Abbreviation is the same as the Table 1; NS = non-significant

### Comparisons between high and low variability of 24 h–SBP groups in participants with OSA

Participants with OSA were separated into two groups based on the median value of weighted 24 h–SD of SBP, and as presented in the Table 4, compared to the low variability group, participants in the high variability group were more elderly, had higher mean 24 h-, daytime- and nighttime-SBP, and mean weighted 24 h, daytime- and nighttime-SD of SBP (*P* < 0.05 for all comparisons). The mean AHI (13.8 ± 7.6 vs 9.9 ± 4.5) was also significantly different. In addition, the prevalence of CVD and the proportion of participants using ACEI/ARB were also significantly higher in the high versus low variability groups.

**Table 4:** Comparisons between high and low variability of 24 h–SBP groups in participants with OSA

Variables	Low variability (*n* = 81)	High variability (*n* = 81)
Age (years)	50 ± 14	55 ± 16*
Male, n (%)	52 (64.2)	54 (66.4)
BMI (kg/m^2^)	27.3 ± 5.7	28.2 ± 6.9
Current smoking, n (%)	38 (46.9)	40 (49.4)
Clinic-SBP (mm Hg)	142 ± 17	143 ± 18
Clinic-DBP (mm Hg)	85 ± 10	86 ± 10
Clinic-HR (bpm)	78 ± 9	79 ± 13
FPG (mmol/L)	5.5 ± 0.5	5.6 ± 0.4
TC (mmol/L)	5.1 ± 0.5	5.2 ± 0.4
TG (mmol/L)	1.8 ± 0.7	1.9 ± 0.8
HDL-C (mmol/L)	1.1 ± 0.5	1.1 ± 0.4
LDL-C (mmol/L)	3.4 ± 0.5	3.3 ± 0.6
Cr (umol/L)	70.6 ± 13.5	72.2 ± 13.9
Hs-CRP (mg/dL)	8.8 ± 3.0	10.2 ± 4.6
24 h–SBP (mm Hg)	132 ± 13	137 ± 15*
24 h–DBP (mm Hg)	78 ± 10	79 ± 12
24-HR (beat per minute)	72 ± 12	74 ± 13
Weighted 24 h–SD of SBP (mm Hg)	12.6 ± 3.7	16.0 ± 4.9*
Weighted 24 h–SD of DBP (mm Hg)	7.4 ± 1.5	8.0 ± 2.2
Daytime-SBP (mm Hg)	137 ± 11	143 ± 18*
Daytime-DBP (mm Hg)	81 ± 11	85 ± 14
Daytime-HR (beat per minute)	77 ± 12	80 ± 15
Weighted daytime-SD of SBP (mm Hg)	14.7 ± 4.0	17.9 ± 5.2*
Weighted daytime-SD of DBP (mm Hg)	8.1 ± 1.6	8.5 ± 2.3
Nighttime-SBP (mm Hg)	128 ± 11	133 ± 14*
Nighttime-DBP (mm Hg)	73 ± 6	77 ± 8
Nighttime-HR (beat per minute)	64 ± 7	68 ± 9
Weighted nighttime-SD of SBP (mm Hg)	10.8 ± 1.5	13.9 ± 2.1*
Weighted nighttime-SD of DBP (mm Hg)	6.5 ± 1.0	7.2 ± 1.3
AHI (events/h)	9.9 ± 4.5	13.8 ± 7.6*
CVD, n (%)	31 (38.3)	35 (43.2)*
Anti-platelet agent, n (%)	34 (42.0)	34 (42.0)
Statins, n (%)	29 (35.8)	30 (37.0)
ACEI/ARB, n (%)	58 (71.6)	61 (75.3)*
CCB, n (%)	17 (21.0)	16 (19.8)
Diuretic, n (%)	26 (32.1)	26 (32.1)
Beta-blocker, n (%)	18 (22.2)	37 (23.5)
Alpha-blocker, n (%)	5 (6.2)	5 (6.2)
Anti-HTN medications number	2.3 ± 0.7	2.6 ± 0.9

Abbreviation is the same as the Table 1; * *P* < 0.05 vs low variability group

### Association between weighted 24 h–SD of SBP and CVD in OSA patients

Logistic regression analysis was applied to evaluate the association between weighted 24 h–SD of SBP and composite CVD in OSA patients. As presented in the Table 5, in the unadjusted model, per 1-SD increment 24 h–SD of SBP was associated with nearly 2-fold increased prevalence of CVD, and after gradually adjusted for potential covariates, per 1-SD increment in weighted 24 h–SD of SBP remained associated with 21% increased prevalence of CVD even after adjusted for clinic-SBP and 24 h–SBP. In addition, the association of weighted 24 h–SD of SBP and prevalent CVD in non-OSA patients was also evaluated and no statistical significance was observed after adjusted for 24 h–SBP (odds ratio 1.05, and 95% confidence interval 0.94–1.11, *P* = 0.127).

**Table 5:** Association of weighted 24 h–SD of SBP and prevalent CVD in OSA patients

	OR and 95% CI	*P* value
Unadjusted	1.97 (1.63–2.18)	<0.001
Model 1	1.75 (1.38–1.92)	0.004
Model 2	1.42 (1.19–1.64)	0.018
Model 3	1.21 (1.09–1.37)	0.035

OR = odds ratio; CI = confidence interval; model 1 = adjusted for age and male gender; model 2 = model 1 + further adjusted for smoking, body mass index, fasting plasma glucose, total cholesterol, high-sensitivity C-reactive protein, apnea-hypopnea index and clinic-SBP; model 3 = model 1+ model 2 + further adjusted for 24 h–SBP

## Discussion

The principal findings of our current study include: first, patients with newly diagnosed OSA have higher 24 h–SBP variability compared to those without OSA, and the potential determinants of 24 h–SBP variability includes age, prevalent CVD and OSA, usage of ACEI/ARB, CCB and diuretic; second, in patients with OSA, higher 24 h–SBP variability is associated with higher prevalence of CVD, even after adjusted for traditional risk factors including clinic-SBP and 24 h–SBP.

In recent years, numerous observational studies and meta-analysis have consistently showed that increased SBP variability was associated with cardiovascular events. For example, Ohkuma et al. [8] conducted a post-hoc analysis of the ADVANCE trial, and they found that in patients with diabetes mellitus, visit-to-visit variability in SBP predicted cardiovascular events and mortality; in addition, addition of visit-to-visit variability in SBP improved predictive value of traditional CVD risk model. Okada et al. [9] reported that high visit-to-visit variability in BP was associated with cardiac diastolic dysfunction and carotid atherosclerosis. In a cohort study, Muntner et al. [7] observed that higher visit-to-visit BP variability predicted CVD and mortality. In a systemic review and meta-analysis [10], Diaz et al. reported that for each 5 mmHg increased SD of SBP was associated with 17%, 27%, 20% and 22% of higher risk of stroke, CHD, cardiovascular mortality and all-cause mortality. Consistent to previous reports, our current study also showed that higher SBP variability as assessed by 24 h–ABPM was independently associated with prevalence of CVD even after adjusted for traditional risk factors including age, male gender, smoking, total cholesterol, clinic-SBP and 24 h–SBP. However, compared to prior studies, our current study enrolled participants with newly-diagnosed OSA, in whom haven’t been sufficiently evaluated before. In addition, prior studies used visit-to-visit variability in SBP, and our current study used 24 h–SBP variability as assessed by 24 h–ABPM which provided novel insights into the association of variability of 24 h–SBP and prevalence of CVD in patients with OSA.

Interestingly and importantly, as anticipated, we observed that patients with newly-diagnosed OSA, compared to those without OSA, had significantly higher 24 h- SBP variability. As we have already mentioned before that patients with OSA commonly have increased sympathetic nerve output, potential endothelial dysfunction and arterial stiffness which together render them at increased probability of short-term and long-term BP fluctuation. In addition, we also observed that in the linear regression analysis, age, presence of CVD and OSA and usage of ACEI/ARB were potential determinants of BP fluctuation. Notably, aging, presence of CVD and OSA are all independently associated with arterial stiffness and endothelial dysfunction, which in turn may cause BP fluctuation. Consistent to prior reports [19–22], we also observed that usage of ACEI/ARB seemed to increase BP variability while CCB and diuretic tended to decrease BP variability. The underlying mechanisms are still unclear yet. However, the data from our current study indicate that in OSA patients with hypertension, it is clinically relevant to monitor and compare BP variability with different classics of anti-hypertensive medications.

There are several limitations of our current study. First of all, the inherent limitation in terms of cross-sectional design could not allow us to draw causal relationship; however, our current study provides novel insight into the association of 24 h–SBP variability and prevalence of CVD in patients with OSA; second, in contrast to using visit-to-visit variability in clinic or home BP, we used 24 h–ABPM to evaluate the 24 h–SBP variability and therefore, the findings from our current study should not extrapolated to those using visit-to-visit variability in home or clinic BP. Last but not least, participants enrolled in our current study were all newly-diagnosed OSA and without diabetes mellitus or chronic kidney disease. Therefore, the findings should not be extrapolated to these populations groups.

## Conclusion

In summary, patients with newly-diagnosed OSA have higher 24 h–SBP variability compared to those without OSA, and age, prevalent CVD and OSA are the potential determinants of 24 h–SBP variability, while anti-hypertensive medications may also influence 24 h–SBP variability. In patients with OSA, those with high 24 h–SBP variability appear to have higher prevalence of CVD compared to their OSA counterparts with low 24 h–SBP variability. Future randomized controlled trials are warranted to evaluate whether improving OSA could contribute to improving 24 h–SBP variability and cardiovascular outcomes.

## Abbreviation

(24 h–ABPM): 24 h ambulatory blood pressure monitoring
(ACEI/ARB): Angiotensin converting enzyme inhibitor/angiotensin receptor blocker
(AHI): Apnea-hypopnea index
(BMI): Body mass index
(CCB): Calcium channel blocker
(CHD): Coronary heart disease
(CPAP): Continuous positive airway pressure
(Cr): Creatinine
(CVD): Cardiovascular disease
(DBP): Diastolic blood pressure
(FPG): Fasting plasma glucose
(Hs-CRP): High-sensitivity C-reactive protein
(OSA): Obstructive sleep apnea
(PSG): Polysomnography
(SaO_2_): Saturation of arterial oxygen
(SBP): Systolic blood pressure
(SD): Standard deviation
